# Accuracy of Capillary Hemoglobin Measurements for the Detection of Anemia among U.S. Low-Income Toddlers and Pregnant Women

**DOI:** 10.3390/nu9030253

**Published:** 2017-03-09

**Authors:** Safia Boghani, Zuguo Mei, Geraldine S. Perry, Gary M. Brittenham, Mary E. Cogswell

**Affiliations:** 1 Division of Nutrition, Physical Activity, and Obesity, National Center for Chronic Disease Prevention and Health Promotion, Centers for Disease Control and Prevention (CDC), Atlanta, GA 30341, USA; safia.b@gmail.com; 2 Division of Adult and Community Health, National Center for Chronic Disease Prevention and Health Promotion, Centers for Disease Control and Prevention (CDC), Atlanta, GA 30341, USA; gperry3943@bellsouth.net; 3 Division of Pediatric Hematology, Department of Pediatrics, Columbia University College of Physicians and Surgeons, New York, NY 10032, USA; gmb31@columbia.edu; 4 Division for Heart Disease and Stroke Prevention, National Center for Chronic Disease Prevention and Health Promotion, Centers for Disease Control and Prevention, Atlanta, GA 30341, USA; mec0@cdc.gov

**Keywords:** anemia, hemoglobin, iron deficiency, children, pregnant women

## Abstract

The aim of this study is to evaluate the accuracy of capillary hemoglobin (Hb) measurements in detecting anemia among low-income toddlers (aged 12–35 months) and pregnant women. In analyses of data among toddlers from Kansas City (*n* = 402) and St. Louis, Missouri (*n* = 236), and pregnant women at <20 weeks gestation from Cleveland, Ohio (*n* = 397), we compared subjects’ anemia status based on capillary Hb concentrations in finger puncture samples as measured by the HemoCue system with their anemia status based on venous Hb concentrations as measured by the HemoCue and Coulter Counter. The sensitivity of capillary blood analyses in identifying cases of anemia was 32.8% (95% Confidence Intervals (CI): 21.0%–46.3%), among Kansas City toddlers, 59.7% (95% CI: 45.8%–72.4%) among St. Louis toddlers, and 66.7% (95% CI: 46.0%–83.5%) among pregnant women in Cleveland; the corresponding specificities were 97.7%, 86.6%, and 96.7%, respectively. The correlation between HemoCue and Coulter Counter measurements of venous Hb (0.9) was higher than that between HemoCue measurements of capillary and venous blood (0.8). The results show that Hb measurements of capillary blood with HemoCue were not optimal for determining the anemia status of toddlers and pregnant women.

## 1. Introduction

Pregnant women and toddlers in the United States are at risk for anemia (i.e., low hemoglobin (Hb) concentration) related to iron deficiency [1,2,3]. Between 2007 and 2010, the prevalence of iron deficiency, anemia, and iron deficiency anemia among US children 1–5 years was 7.1, 3.2%, and 1.1%, respectively [4]. An accurate means of measuring Hb concentration is important for clinical diagnosis of anemia, research, and public health practice. The detection of anemia is the most common method used to screen individuals for iron deficiency [5]. Because of the feasibility and low cost of measuring Hb concentrations, Hb measurements are often used to estimate the prevalence of iron deficiency anemia, as well as to indicate the iron status of a population and the success of interventions to increase iron levels [5,6].

The accuracy of capillary sampling methods for the detection of anemia in US toddlers and pregnant women is unclear. In previous studies, the accuracy of Hb measurements of capillary blood conducted with portable hemoglobinometers (compared with Hb measurements of venous blood conducted with a reference method such as Coulter Counter (Beckman Coulter, Indianapolis, IN, USA), a device used to measure Hb using the cyanmethohemoglobin spectrophotometry method), varied within and across populations [7,8,9,10,11,12,13,14,15,16,17,18,19,20,21,22,23,24,25,26]. Furthermore, only one of these studies included toddlers (children aged 12–35 months), and the authors of that study did not provide estimates of the accuracy of capillary sampling for the detection of anemia specifically among toddlers [19]. Toddlers may present particular challenges for capillary sampling because they are less likely to comply with blood-collection procedures than adults or older children. In addition, only two of the studies of capillary sampling included pregnant women [7,20], and the small sample size of those studies (*n* = 29 and 108) precluded an examination of the sensitivity and specificity of Hb measurements of capillary blood relative to reference method Hb measurements. To help determine the accuracy of Hb measurements of capillary blood and of anemia diagnoses among toddlers and pregnant women based on these measurements, we compared Hb values (and corresponding anemia status determinations) based on analyses of capillary samples with portable hemoglobinometers to reference Hb values (and corresponding anemia status determinations) based on analyses of venous blood with Coulter Counters. 

## 2. Materials and Methods 

We analyzed data collected in three previous studies involving participants in the Supplemental Nutrition Program for Women, Infants, and Children (WIC): one at two clinics in Kansas City, MO, USA (Study A; *n* = 413 toddlers), one at two clinics in St. Louis, MO, USA (Study B; *n* = 213 toddlers), and one at a clinic in Cleveland, OH, USA (Study C; *n* = 397 pregnant women). The original study protocols were approved by the institutional review board (IRBs) at each study site and by the Centers for Disease Control and Prevention (CDC) Office on Human Subjects. All participating women and the parents of all participating toddlers provided written informed consent. A brief description of these studies follows.

Study A and Study B: The primary objective of these two studies was to investigate the etiology of anemia among children aged 12 to 35 months. Phlebotomists trained in venipuncture and capillary blood sampling techniques collected a 2.0-mL venipuncture blood sample from each participant. They then sent part of the sample to a local hospital laboratory, which determined the Hb concentration using an automated Coulter Counter. The phlebotomists also filled two HemoCue cuvettes (Hemocue America, Brea, CA, USA) with other parts of the sample, determined the Hb concentration in each, and averaged the results. In addition, they collected capillary blood samples from participants’ fingertips and analyzed these samples with the HemoCue method at the clinic. In Kansas City, 452 toddlers presented between April 2001 and September 2002, and, in St. Louis, 305 toddlers presented between January 2002 and April 2003.

Study C: The main objective of this study was to estimate the impact of iron supplementation for pregnant women on maternal and infant outcomes (15). As part of this study, a 5-mL venipuncture sample was collected by a trained phlebotomist and analyzed in a hospital laboratory with use of an automated Coulter Counter and HemoCue. In addition, a cuvette from a separate container was filled with a second drop of capillary blood from participants’ fingertips for analysis by the HemoCue method at the clinic. Data were collected from 513 pregnant women; however, we restricted our analysis to data from women who had been pregnant less than 20 weeks when they enrolled in WIC since, after 20 weeks, the women were randomly assigned to receive iron capsules or placebo as part of the study.

We excluded from our analyses all study participants with missing Hb concentration data for capillary blood specimens analyzed with the HemoCue method or venous blood specimens analyzed with the Coulter Counter. We also excluded WIC participants in Kansas City and Cleveland who were missing Hb concentration data for venipuncture specimens analyzed with HemoCue, but we did not do so for St. Louis participants because only a minority of children had venous blood samples analyzed by the HemoCue method. We excluded 39 children from Kansas City and 67 from St. Louis because they did not have venous blood drawn, as well as 116 women from Cleveland because venous blood was not collected from them until after they had enrolled in the study. We also excluded participants from all three studies if they had implausible Hb values (less than 4 g/dL or more than 16 g/dL (*n* = 2)) or their records did indicate their age or sex (*n* = 23). Our analytic sample after all exclusions consisted of 413 toddlers from Kansas City, 213 toddlers from St. Louis, and 397 pregnant women from Cleveland. 

## 3. Statistical Analysis

We estimated the means, standard deviations, and coefficients of variation in Hb values for each blood sampling and analytic method by study site. For Kansas City participants, we averaged the first two Hb values in venous samples tested by the HemoCue method to determine their venous HemoCue Hb concentration. As noted previously, we did not include results for venous blood samples from St. Louis participants analyzed by the HemoCue method because only a minority of them had these measurements. As also noted previously, we used age-specific Hb cut-points to determine the anemia status of children (11.0 g/dL for children aged 12–23 months and 11.1 g/dL for children aged 23–35 months) and trimester-specific cutpoints to determine the anemia status of pregnant women (11.0 g/dL for women in their first trimester and 10.5 g/dL for women in their second trimester) (5). To evaluate the performance of the various sampling and analytic methods, we calculated the sensitivity and specificity of the methods for detecting anemia relative to determinations of anemia based on the reference method (venous blood analyzed with the Coulter Counter). We used Spearman correlation coefficient to assess correlations in Hb values produced by the various sampling and analytic methods and a paired Student’s *t*-test to compare mean Hb values. For each participant, we compared Hb results from each method with those obtained by Coulter Counter measurements of venous blood and report the proportion of participants for whom the difference in Hb values divided by the mean of the Hb values obtained with the two methods was less than 5%. We also used the mean Hb value of the two methods as the *x*-axis values in Bland–Altman scatter plots (16), which we used to depict differences in Hb concentrations by type of analytic method (7, 16). We considered *p*-values < 0.05 to be indicative of statistically significant differences. All analyses were conducted with SAS version 9.2 (SAS Institute INC, Cary, NC, USA).

## 4. Results

**Figure 1:**
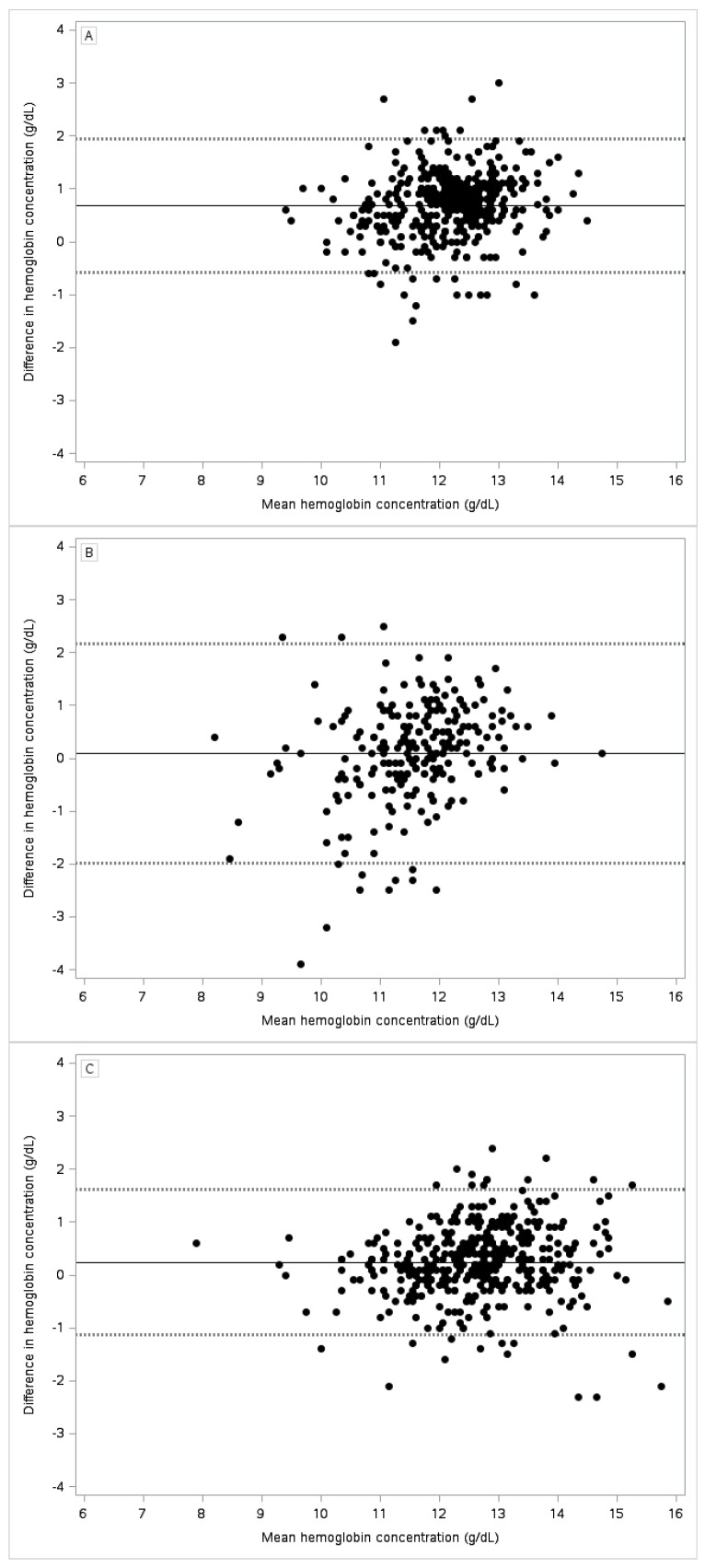
Bland–Altman scatter plots of the difference in hemoglobin concentration from capillary blood analyzed with HemoCue vs. venous blood analyzed with Coulter Counter by the mean of the estimates. Dashed lines represent 95% limits of agreement. Panel (**A**) Kansas City (*n* = 413), Panel (**B**) St. Louis (*n* = 213), and Panel (**C**) Cleveland (*n* = 397).

Demographic characteristics of the three populations are given in Table 1. The anemia prevalence was significantly lower among toddlers in Kansas City than among toddlers in St. Louis and pregnant women in Cleveland (Table 2). Capillary blood analyzed with HemoCue yielded higher Hb values than venous blood analyzed with Coulter Counter among toddlers in Kansas City (Figure 1, Panel A) but not among toddlers in St. Louis (Panel B) or among pregnant women in Cleveland (Panel C). Similarly, the mean paired differences between Hb values derived from capillary blood analyzed with HemoCue and Hb values derived from venous blood analyzed with Coulter Counter varied by study site (from 0.07 g/dL in St. Louis to 0.69 g/dL in Kansas City), although the variations were not statistically significant.

**Figure 2:**
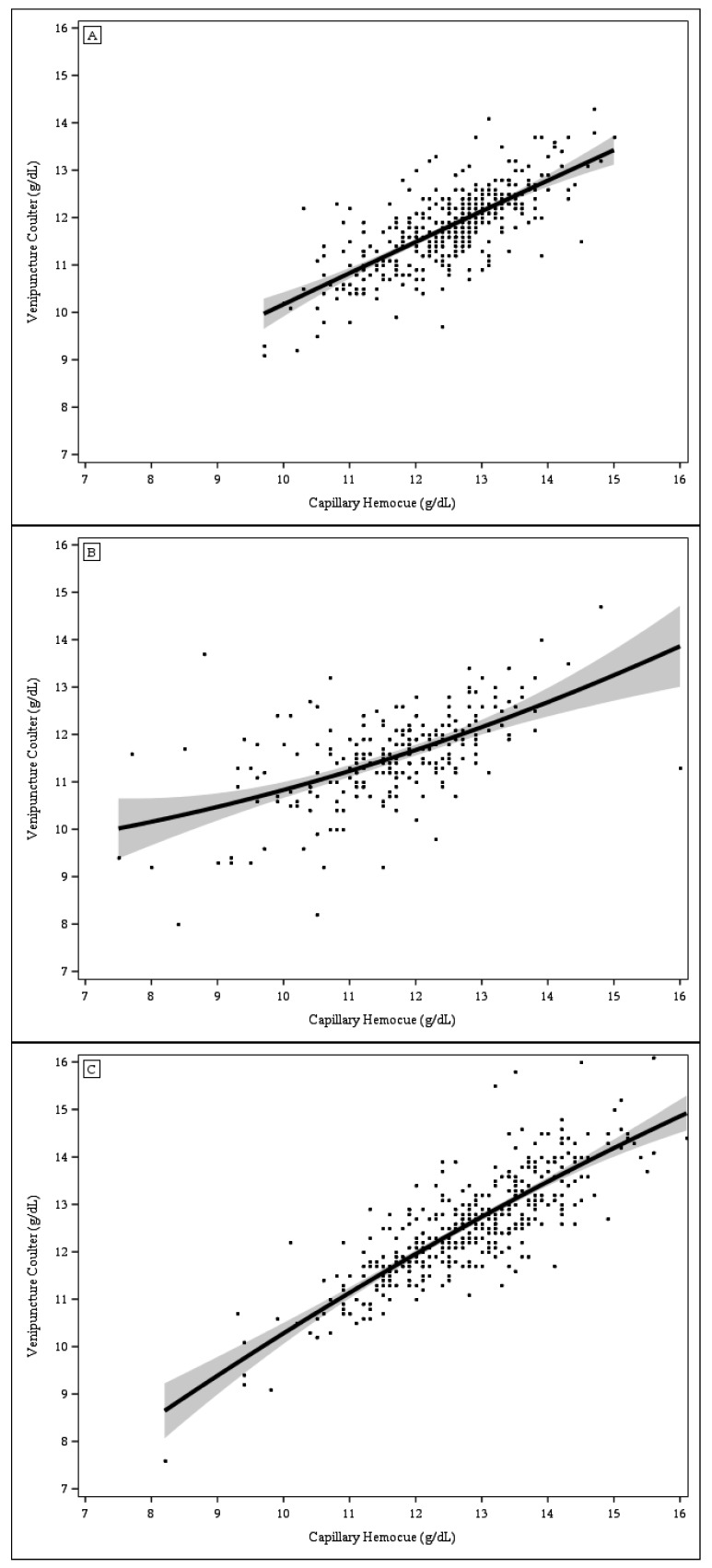
Scatter plots of hemoglobin concentration from capillary blood analyzed with HemoCue vs. venous blood analyzed with Coulter Counter. The solid line represents the mean regression line and the grey areas represent the 95% confidence limits. (**A**) Kansas City (*n* = 413); (**B**) St. Louis (*n* = 213); and (**C**) Cleveland (*n* = 397).

The correlations between Hb concentrations from capillary blood analyzed with HemoCue and those from venous blood analyzed with Coulter Counter were lower among St. Louis toddlers (0.54) than among Kansas City toddlers (0.74) or Cleveland women (0.82). The scatter plots of Hb concentrations from capillary blood analyzed with HemoCue vs. Hb concentrations from venous blood analyzed with Coulter Counter (Figure 2, Panels A–C) also indicate a greater discordance in Hb values among St. Louis toddlers, particularly among those with Hb values <9.0 g/dL. No study participants other than those from St. Louis had Hb values <9.0 g/dL. The sensitivity of anemia detection based on Hb levels in capillary samples analyzed with the Coulter Counter was substantially lower in Kansas City (32.8%) than in St. Louis (60.4%) or Cleveland (66.7%), (*p* = 0.0023), whereas the specificity was lower in St. Louis (85.6%) than in Kansas City (97.7%) or Cleveland (98.1%) (*p* < 0.0001 for both comparisons).

The mean paired differences in Hb values between venous blood analyzed with HemoCue and venous blood analyzed with Coulter Counter was 0.38 g/dL (−0.35, 1.12) among Kansas City toddlers and −0.20 g/dL (−1.27, 0.86) among pregnant women in Cleveland (Table 2). The correlations in Hb values produced by these two methods were 0.90 and 0.88, respectively. The difference in Hb values divided by the mean value of the two methods was less than 5% among 81.6% of Kansas City toddlers and 86.2% of Cleveland women. The anemia detection rate for HemoCue analysis of venous blood was 51.7% among toddlers in Kansas City and 92.6% among pregnant women in Cleveland, (*p* = 0.0002). The corresponding specificities were 99.4% and 96.7% (*p* = 0.0104), respectively. 

The mean paired difference between Hb values obtained by HemoCue analysis of capillary samples and those obtained by HemoCue analysis of venous samples was 0.31 g/dL among Kansas City toddlers and 0.44 g/dL among Cleveland women. Neither difference was significant (Table 3). The correlations between Hb values from HemoCue analyses of capillary samples and HemoCue analyses of venous samples in these two populations were 0.72 and 0.81, respectively, lower than the correlations between Hb results obtained by different analytic methods. The difference in Hb values divided by the mean value of the two methods was less than 5% among 59.6% of Kansas City toddlers and 55.9% of Cleveland women. 

## 5. Discussion

We found that Hb measurements of capillary blood with HemoCue were not optimal for detecting anemia among toddlers and pregnant women. The sensitivity of anemia detection with this method was less than 70% in all three study groups, and the specificity was less than 90% among toddlers in St. Louis. Intraperson variation in Hb measurements was significantly larger by sampling method (capillary vs. venous) than by analytic method (HemoCue vs. Coulter Counter). In addition, our results suggest a systematic upward bias in Hb values derived from analyses of capillary samples in Kansas City but not in St. Louis or Cleveland.

In previous studies [17,18,19,23,24], the sensitivity of anemia diagnoses based on capillary blood analyses with HemoCue ranged from 56% to 95%, and the specificity of these diagnoses ranged from 93% to 97%, respectively, when compared with diagnoses based on cyanmethohemoglobin spectrophotometry (reference) methods. However, we found the sensitivity of capillary blood analyses to be lower than 71% at all our study sites and the specificity to be lower than 93% among toddlers in St. Louis. As noted previously, we found only one study that used the accuracy of capillary blood analysis in identifying people with anemia that included toddlers [19]. Although the small sample size of that study precluded an analysis specifically among toddlers, the authors did note that the concordance between Hb values obtained with capillary blood analyses and values obtained with venous blood analyses was lower among children aged 0.5–15 years than among adults aged 17–73 years. 

Our results, which fall within or close to the range of results from some previous studies, including those among children and pregnant women [15,19,20,27,28], indicate that assessments of Hb concentrations in capillary blood are higher than assessments of Hb concentrations in venous blood when samples are analyzed with the same method. However, in three other studies, one among children [29] and two among adults [23,30], Hb concentrations in capillary blood were found to be lower (−0.1 to −0.5 g/dL) than concentrations in venous blood when analyzed with the same method [23,29,30]. In Cleveland, but not Kansas City, the differences between Hb concentrations in venous blood and Hb concentrations in capillary blood also may be related to use of different machines and containers of cuvettes in analyses of capillary blood than in analyses of venous blood. Despite this difference in study procedures, the mean paired difference by sampling method in Cleveland was similar to that in Kansas City. In studies among neonates [30] and adults [31], whole blood and/or lymphocyte counts were found to be higher in capillary samples than in venous samples, but differences in Hb and red blood cell counts were not consistent across these two studies. 

The authors of a previous study [17] suggested that differences in Hb concentration by sampling method may be explained in part by a lack of standardization in capillary sampling. Our finding that the standard deviation (SD) in Hb concentrations for capillary samples measured by HemoCue was significantly larger than that for venous samples measured by Coulter Counter in St. Louis (SD 1.2 g/dL vs. SD 0.9 g/dL), but not at the other two study sites, similarly suggests a problem with sampling standardization at the St. Louis site. In Cleveland and Kansas City, a single phlebotomist collected capillary samples, whereas in St. Louis, capillary samples were collected by multiple phlebotomists. The larger SDs in Hb values (regardless of analytic or sampling method) in Cleveland (1.1–1.2 g/dL) may be related to the between individual biological variation in Hb with the hemodilution of pregnancy. 

We did not find a significant difference between the mean Hb concentration in venous blood as measured with HemoCue and that in venous blood as measured with the Coulter Counter. Results from previous studies have been inconclusive, with the difference in Hb values ranging from 0.6 g/dL to 0.5 g/dL higher [17,18,19,20,23,30,31,32,33,34,35]. Our finding of a high correlation (~0.9 or higher) between Hb concentrations in venous blood analyzed with HemoCue and Hb concentrations in venous blood analyzed by direct cyanmethemolglobin was in general agreement with those from previous studies showing a similarly high correlation [17,18,19,20,32]. Although we found that >80% of study participants had Hb values by each method that were within 5% of the mean value for both, we also found that estimates of the prevalence of anemia based on Hb values determined by the two methods varied substantially. 

Strengths of our study included its relatively large sample size and varied population, which allowed us to assess differences in sampling and analytic methods for measuring Hb between toddlers at two study sites and between toddlers and pregnant women. The variation in the prevalence of anemia across sites allowed us to examine potential bias over a wide range of Hb concentrations.

One of the limitations to our study is that only a minority of children from the St. Louis site had venous blood samples analyzed by the HemoCue method. As a result, we were unable to determine whether differences in Hb measurements at this site were due to a difference in sampling methods or to a difference in analytic methods. In the sub-sample of toddlers who had their Hb concentration measured by HemoCue in both venous and capillary blood samples, the mean concentration in capillary samples was 0.46 g/dL higher (95% Limits of Agreement (LOA), −1.67, 2.60), although not significantly higher. Another study limitation is that missing measurements for some participants introduced a possible selection bias. 

## 6. Conclusions

In summary, our results suggest that analyses of capillary blood samples from toddlers and pregnant women with HemoCue indicate higher Hb concentrations than analyses of venous blood samples with Coulter Counter; as a result, estimates of the prevalence of anemia among toddlers and pregnant women are likely to be lower if based on analyses of capillary samples than if based on analyses of venous samples. However, because of variation in results across participating study sites, we were not able to compensate for differences between Hb concentrations measured in capillary samples and Hb concentrations measured in venous samples by applying a single adjustment factor. Our finding that the apparent accuracy of the HemoCue analytic method varied by clinic suggests a need for more rigorous technician training and ongoing quality control efforts. Our finding that population-based estimates of the prevalence of anemia based on analyses of capillary blood are likely to be lower than the true underlying prevalence of anemia in that population suggests that anemia screening based on analyses of capillary samples should be followed up with confirmatory tests for anemia in venous blood and that estimates of the prevalence of anemia in a given population based on capillary sample analyses should be compared with estimates in a segment of that population based on venous blood analyses to determine if correction for a systematic bias is warranted. 

## Figures and Tables

**Table 1:** Sociodemographic characteristics of participants * by site.

	Site		
Characteristics	Kansas City, Missouri	St. Louis, Missouri	Cleveland, Ohio
Population	Low-income toddlers from two clinics	Low-income toddlers from two clinics	Low-income pregnant women from one clinic
Sample size, *n*	413	213	397
Mean age	22 months	23 months	24 years
Female, %	48	49	100
Race, %	Black—66	Not Available	Black—28
	Hispanic—7		Hispanic—16
	White—15		White—55
	Other—12		Other—1

* Participants were enrollees in the Supplemental Nutrition Program for Women, Infants, and Children (WIC).

**Table 2:** Comparisons of hemoglobin concentration measurements among Women, Infants, and Children (WIC) participants in three previous studies, by sampling and analytic method for data collected between 2002 and 2003.

	Kansas City, *n* = 413			St. Louis, *n* = 213		Cleveland, *n* = 397		
Sampling Method:	Capillary	Venous	Venous	Capillary	Venous	Capillary	Venous	Venous
Analytic Method:	HemoCue	HemoCue	Coulter	HemoCue	Coulter	HemoCue	HemoCue	Coulter
Mean, g/dL	12.5 (10.9, 13.9)	12.2 (10.7, 13.7)	11.8 (10.4, 13.1)	11.6 (9.4, 13.4)	11.5 (9.6, 13.0)	12.8 (10.9, 14.6)	12.4 (10.5, 14.1)	12.6 (910.7, 14.4)
Standard deviation, g/dL	0.9	0.9	0.8	1.2	0.9	1.2	1.1	1.1
Anemic, %	6.8 (4.6, 9.7) ^1^	8.7 (6.2, 11.9)	14.4 (11.0, 18.9)	25.8 (20.1, 32.3)	24.9 (19.2, 30.7)	6.3 (3.9, 8.7)	9.3 (6.5, 12.2)	6.8 (4.3, 9.3)
Mean paired difference ^2^ (95% limits of agreement), g/dL	0.69 ^3^ (−0.55, 1.94)	0.36 (−0.59, 1.31)	Referent	0.07 (−2.01, 2.15)	Referent	0.24 (−1.11, 1.60)	−0.20 (−1.27, 0.86)	Referent
Spearman correlation coefficient	0.72 (0.67, 0.76)	0.89 (0.87, 0.91)	Referent	0.56 (0.46, 0.64)	Referent	0.81 (0.77, 0.84)	0.89 (0.87, 0.91)	Referent
Participants for whom the difference between Two methods divided by the means of two methods <5%, ^4^ %	38.3 (33.6, 43.1)	76.3 (71.9, 80.3)	Referent	52.6 (45.9, 59.3)	Referent	66.3 (61.4, 70.9)	86.2 (82.4, 89.4)	Referent
Sensitivity, ^5^ %	32.8 (21.3, 46.0)	51.7 (38.2, 65.1)	Referent	60.4 (44.1, 71.4)	Referent	66.7 (46.0–83.5)	92.6 (75.7, 99.1)	Referent
Specificity, ^6^ %	97.7 (95.6, 99.0)	97.7 (95.6, 99.0)	Referent	85.6 (79.2, 90.7)	Referent	98.1 (96.1, 99.2)	96.7 (94.4, 98.3)	Referent

^1^ 95% confidence interval (CI) presented in parentheses, unless otherwise specified; ^2^ Difference in hemoglobin measured by the method indicated versus the referent (venous coulter); ^3^ Paired *t*-test, *p* < 0.05; ^4^ Number of participants ((difference between two methods/mean of two methods) < 0.05)/all participants; ^5^ Number anemic by the indicated method and the referent method divided by number anemic by the referent method; ^6^ Number not anemic by the indicated method and the referent method divided by number not anemic by the referent method.

**Table 3:** Three statistical comparisons of hemoglobin concentrations derived from HemoCue analyses of capillary blood samples with concentrations derived from HemoCue analyses of venous blood samples from toddlers in Kansas City and pregnant women in Cleveland.

Statistical Comparisons	Kansas City (*n* = 402)	Cleveland (*n* = 397)
Correlation coefficient	0.72 (0.67, 0.76)	0.81 (0.77, 0.84)
Mean paired difference for capillary minus venous hemoglobin (95% limits of agreement), g/dL	0.33 ^1^ (−0.99, 1.66)	0.44 ^1^ ( 0.96, 1.84)
% of participants for whom the difference in Hb values derived from the 2 methods divided by the mean Hb value of the 2 methods was <5% ^2^	59.6 (71.9, 80.3)	55.9 (50.9, 60.9)

^1^ Paired *t*-test, *p* < 0.05; ^2^ Number of participants ((difference between two methods/mean of two methods) <0.05)/all participants.
